# FusionLite-DETR: A Lightweight Transformer-Based Model for UAV-Based Insulator Defect Detection

**DOI:** 10.3390/s26206537

**Published:** 2026-10-15

**Authors:** Wenlong Fu, Bo Lan, Yahui Shan, Nan Li, Baotong Chi, Zehao Kong

**Affiliations:** 1 Hubei Provincial Key Laboratory for Operation and Control of Cascaded Hydropower Station, China Three Gorges University, Yichang 443002, China; 2 College of Electrical Engineering, China Three Gorges University, Yichang 443002, China; 3 Wuhan Second Ship Design and Research Institute, Wuhan 430064, China

**Keywords:** UAV inspection, insulator, object detection, RT-DETR, VanillaNet

## Abstract

Aiming to overcome the challenges associated with the unmanned aerial vehicle (UAV)-based inspection of transmission line insulators, including the tiny scale of defect regions, irregular morphologies, and low contrast with complex backgrounds, we propose a lightweight insulator defect detection model based on real-time detection transformer (RT-DETR), termed FusionLite-DETR. To improve detection accuracy while maintaining computational efficiency, the proposed model introduces systematic enhancements to RT-DETR in four aspects: the backbone network, feature reconstruction operator, feature fusion module, and loss function. Firstly, the backbone network is reconstructed by integrating deformable large kernel attention (DLKA) into VanillaNet, enabling the model to couple global perception with precise local focusing, thereby significantly enhancing feature extraction for multi-scale defects. Secondly, the defect-aware content-aware reassembly of features (DA-CARAFE), is proposed to replace the conventional upsampling method. It dynamically generates reassembly convolution kernels based on underlying spatial features, achieving semantically aligned feature fusion under lightweight constraints. Meanwhile, a wavelet transform-based C3 (WTC3), is designed. By performing feature representation and interaction across different frequency sub-bands and incorporating ghost-shuffle convolution (GSConv), the module effectively reduces parameter redundancy while preserving critical high-frequency texture information. Furthermore, by introducing the inner minimum point distance intersection over union (Inner-MPDIoU) loss function, the boundary discrepancies are accurately captured by combining auxiliary-box overlap measurement with vertex-based geometric distance constraints, thus effectively mitigating the gradient vanishing problem for distant small targets during regression. FusionLite-DETR achieved mean average precision at an intersection-over-union threshold of 0.50 (mAP50) values of 95.5% and 93.1% on the private multi-scenario insulator defect dataset (PMID) and insulator fault detection dataset IFDD (datasets), respectively, with 19.1 M parameters and a computational cost of 54.8 giga floating-point operations (GFLOPs). Compared with RT-DETR with a resnet-50 backbone (RT-DETR-R50), the proposed model reduced the parameter count and GFLOPs by 54.8% and 57.4%, respectively, demonstrating a favorable balance between detection performance and model complexity.

## 1. Introduction

Serving as the critical mechanical interface between transmission cables and supporting pylons, insulators play an indispensable role in overhead power networks. Continuous subjection to severe weather environments leaves insulators prone to mechanical impairment, thereby undermining the dependable and steady performance of transmission lines [1,2]. Therefore, regular inspection of transmission lines and precise identification of insulator defects are critical for maintaining the long-term stable operation of the power grid [3].

Traditional manual and on-site inspections have long been the standard method for detecting insulator faults. However, these conventional practices are hindered by fundamental shortcomings, characterized by poor time efficiency, high labor intensity, and pronounced subjective variations in the detection results. Moreover, it is significantly constrained by terrain and environmental factors, making it difficult to effectively guarantee both identification accuracy and operational safety [4]. Recently, fueled by the accelerated progress and practical deployment of unmanned aerial vehicle (UAV) remote sensing alongside computer vision algorithms, integrating visual sensors on UAV platforms with advanced computer vision techniques has emerged as an efficient and safe solution for intelligent insulator inspection in the power industry [5,6,7,8].

Existing studies have introduced convolutional neural networks and Transformer-based object detectors into UAV-based insulator inspection, achieving considerable improvements over traditional image-processing methods. Nevertheless, detecting insulator defects in aerial images remains challenging. Such defects generally occupy only a small proportion of the image and often exhibit irregular shapes, weak visual features, and substantial scale variations. Complex backgrounds, illumination changes, and variations in imaging distance may further obscure their appearance. Although real-time detection transformer (RT-DETR) [9] provides an effective end-to-end detection framework with favorable real-time performance, its conventional backbone may introduce computational redundancy, while its feature-fusion architecture may not adequately preserve the spatial and boundary information of small defects.

To address these limitations, we propose FusionLite-DETR, a lightweight insulator defect detection model built upon RT-DETR. Tailored to the practical requirements of UAV-based transmission-line inspection, FusionLite-DETR redesigns the backbone, upsampling operator, feature-fusion structure, and bounding-box regression loss to enhance feature representation and improve the detection and localization accuracy of small defects. The main contributions of this paper are summarized as follows:

(1) VanillaNet with deformable large-kernel attention (VanillaNet-DLKA) backbone network: We reconstruct the backbone network by integrating deformable large kernel attention (DLKA) [10] into VanillaNet [11]. This mechanism leverages the expansive receptive field of large kernels and the precise local focusing of deformable sampling, significantly enhancing the capture of multi-scale features while reducing computational overhead through the minimalist VanillaNet design.
(2) Defect-aware content-aware reassembly of features (DA-CARAFE): A novel upsampling operator is proposed to replace the standard interpolation in the feature pyramid network [12]. DA-CARAFE utilizes underlying spatial information to predict reassembly kernels, enabling semantically aligned feature fusion within predefined neighborhoods while maintaining a lightweight profile.
(3) Feature fusion module: An innovative feature fusion module, wavelet transform C3 (WTC3), is designed based on the Wavelet Transform (WT) [13]. By incorporating feature representations across different frequency sub-bands, this module replaces the conventional RepC3 [14] structure. This modification optimizes the multi-branch fusion process, reducing parameter redundancy while preserving critical structural information. Additionally, ghost-shuffle convolution (GSConv) [15] is integrated to replace standard convolutions, achieving a balance between reducing computational load and maintaining robust feature representation.
(4) Inner minimum point distance intersection over union (Inner-MPDIoU) loss function: The original loss function is substituted by Inner-MPDIoU, which incorporates a scaling parameter to regulate auxiliary boxes. By calculating geometric distances between the corner points of the predicted and actual boxes, it precisely captures boundary discrepancies. The auxiliary-box overlap mechanism provides stricter boundary supervision for small targets, effectively mitigating the gradient vanishing issue and improving localization precision for distant, small insulator defects.

## 2. Related Work

### 2.1. Traditional and Convolutional-Neural-Network-Based Insulator Defect Detection

Traditional computer vision-based insulator image recognition methods predominantly depend on hand-engineered descriptors, notably the histogram of oriented gradients (HOG) [16] and the scale-invariant feature transform (SIFT) [17]. These methods typically identify target regions through feature extraction, feature point matching, and spatial constraint localization, thus enabling insulator recognition and detection. For instance, Tang et al. [18] proposed a multi-feature fusion approach that assigns gradient weighting to the global texture feature vectors derived from the HOG algorithm. By performing radial gradient transformations to obtain improved HOG features, this approach effectively enhances the representation capability for insulator detection. Zhai et al. [19] proposed a fault detection method utilizing saliency and adaptive morphology, with the goal of investigating the capabilities of both techniques. Xu et al. [20] simulated electric field distributions on insulator shed surfaces to extract relevant features, which were then input into a machine learning model for insulator fault classification. However, these methods share a common limitation in that they exhibit insufficient adaptability to complex real-world environments and are highly sensitive to lighting variations, background interference, and occlusions. Consequently, their detection accuracy and robustness often fail to meet the stringent requirements for insulator defect identification in practical engineering applications. More broadly, recent studies have shown that degraded visual sensing can affect predictive reliability in automated infrastructure inspection, highlighting the importance of considering sensing quality in practical applications [21].

Driven by ongoing advancements in deep learning technology, convolutional neural networks (CNNs) [22] have demonstrated significant advantages in feature representation capabilities, gradually replacing traditional hand-crafted feature design methods, and are widely applied in computer vision-related tasks. As one of the key research directions, current object detection paradigms are generally classified as either one-stage or two-stage models. Two-stage approaches, represented by region-based convolutional neural network (R-CNN) [23], Fast R-CNN [24], Mask R-CNN [25], and Faster R-CNN [26], perform object detection through a sequential process that includes region proposal generation, followed by object classification and bounding box regression. Specifically, these methods first generate a set of candidate regions from an input image, then extract features from these regions, and finally perform category classification and bounding box regression to achieve object recognition and localization.

Alternatively, architectures like the you only look once (YOLO) and single shot multibox detector (SSD) series [27,28] function as single-stage networks, eliminate the intermediate step of region proposal generation, and adopt an end-to-end inference architecture. These methods directly encode features from the input image and perform regression, thereby simultaneously producing defect category information and spatial location coordinates. For example, Tao et al. [29] proposed a cascaded CNN framework for localizing insulators and identifying faults. Chen et al. [30] constructed multi-resolution feature maps and incorporated feature fusion within a lightweight Faster R-CNN framework to enhance detection performance for multi-scale objects. Xin et al. [31] fused shallow and deep feature information within the SSD framework, thereby improving feature expressiveness and detection accuracy for small targets. Kang et al. [32] introduced a dual-stage approach for insulator recognition, utilizing a deep denoising autoencoder. For UAV-acquired images, Souza et al. [33] achieved insulator defect classification using a hybrid YOLO approach. Yi et al. [34] improved YOLOv5 by introducing a SlimNeck lightweight structure, which enhanced inference efficiency while maintaining detection accuracy. Although the aforementioned CNN-based detection methods have achieved significant progress in insulator defect detection, they still have inherent limitations in processing global context information, capturing long-range dependencies, and handling the high variability of defect morphologies, and most still rely on complex anchor box designs or post-processing operations.

### 2.2. Transformer-Based Object Detection

In recent years, as the advantages of Transformers [35] in visual representation modeling and global dependency capture have become increasingly prominent, end-to-end architectures have become a prominent trend in object detection. Serving as foundational work in this direction, the DETR [36] algorithm models the detection process as a direct set prediction task, generating a prediction set in parallel through a preset number of object queries and employing the Hungarian algorithm to implement exclusive sample assignment. This mechanism reduces the reliance on hand-crafted post-processing operations like anchor generation and non-maximum suppression, constructing a concise detection framework that supports end-to-end optimization. To address the inherent challenges of detection transformer (DETR), such as computational overhead, matching instability, and sparse supervision signals, subsequent research has introduced in-depth improvements across multiple dimensions. For example, Deformable DETR [37] introduces multi-scale features and deformable attention to enable sparse aggregation around reference points, thereby mitigating the high computational costs of global interactions and enhancing small object detection. Regarding query modeling, Conditional DETR [38] decouples decoder queries into content and spatial components to constrain the attention response range and reduce reliance on high-quality content embeddings. Additionally, to address the sparse supervision signals caused by strict one-to-one matching in DETR, denoising DETR (DN-DETR) [39] introduces a denoising training mechanism that reconstructs noisy ground-truth boxes and labels to increase training signal density. Building on this, DETR with improved denoising anchor boxes (DINO) [40] integrates contrastive denoising, dynamic anchor boxes, and mixed query selection for more robust end-to-end learning. Recently, D-FINE [41] has reformulated bounding box regression into an iterative refinement of fine-grained distributions and employed self-distillation to enhance both localization precision and shallow feature learnability. Additionally, to address the sparse supervision signals caused by strict one-to-one matching in DETR, DETR with improved matching (DEIM) [42] addresses sparse supervision through dense one-to-one matching and a matchable-aware loss, increasing positive samples while suppressing matching noise to improve convergence and detection performance. Despite significant advancements in addressing slow convergence and unstable matching in the DETR framework, most of the current methods are still primarily evaluated on general-purpose datasets like common objects in context (COCO), focusing on general object detection tasks. Therefore, the generalization, robustness, and engineering adaptability of these methods in industrial vision and defect detection scenarios, which exhibit significant domain differences, have not been systematically assessed, and their practical applicability remains inadequately validated.

## 3. Materials and Methods

### 3.1. Inspection System Workflow

**Figure 1:**
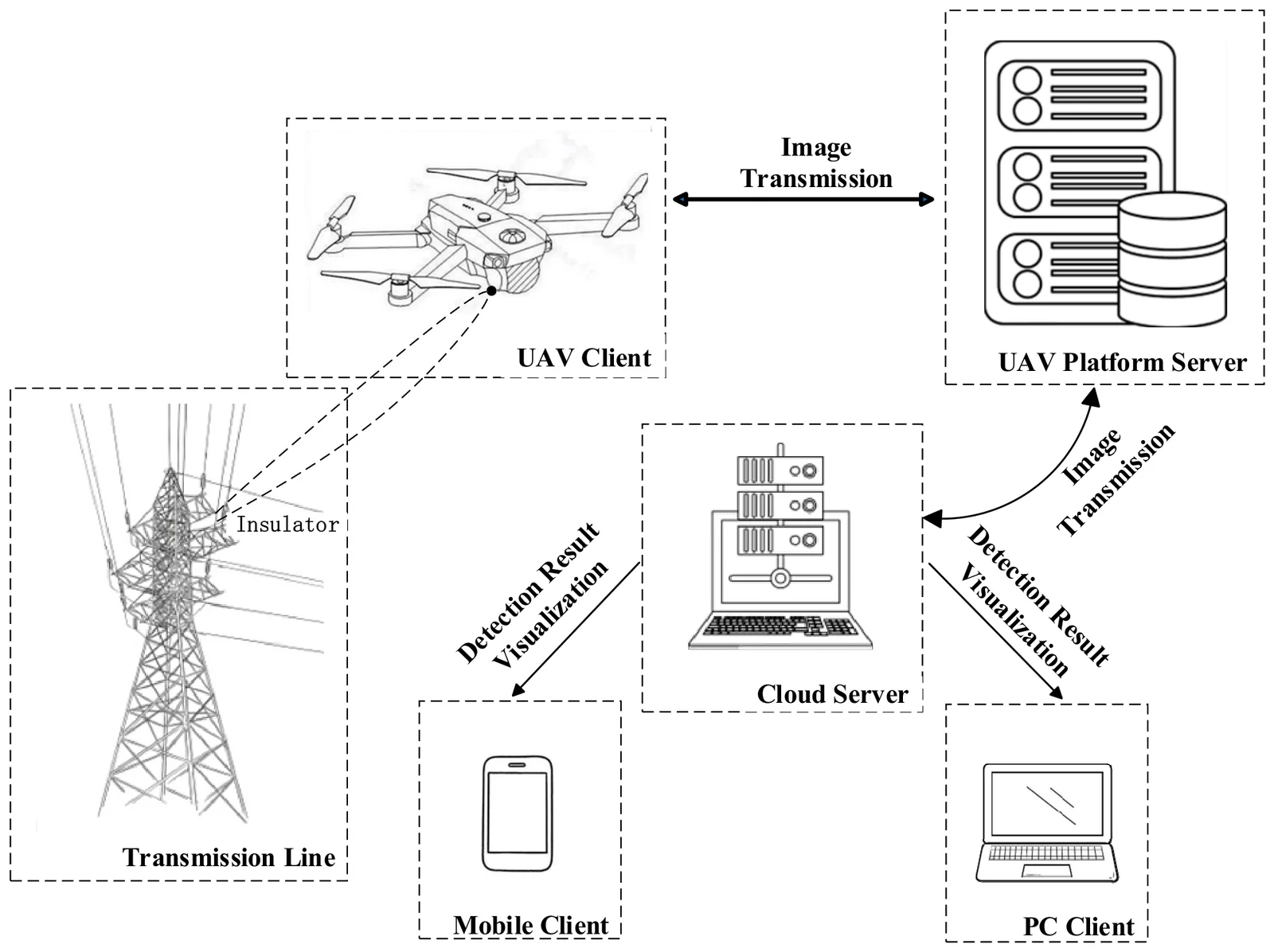
Three-terminal collaborative architecture.

In the practical inspection of power transmission line insulators, the inspection mode employs a three-terminal collaborative architecture. First, UAVs capture insulator image data and transmit it to a platform for storage. Then, the service end retrieves this data from the platform and processes the image data. Finally, the processed data are delivered to the client end for visualization. The detailed inspection workflow is depicted in Figure 1. The deployment of the proposed insulator detection algorithm at the service end has the potential to improve the efficiency of the overall inspection workflow.

### 3.2. RT-DETR Baseline

**Figure 2:**
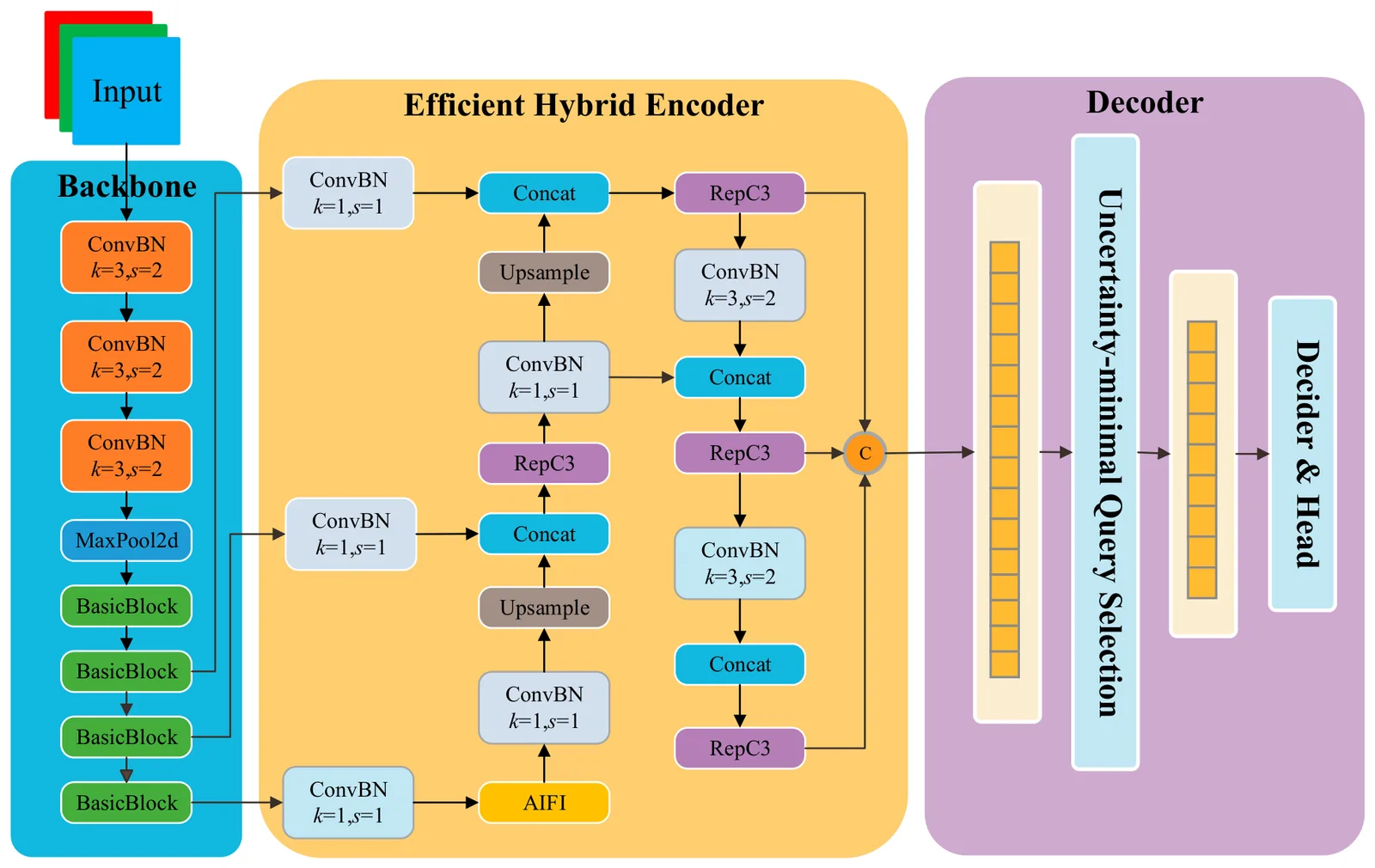
RT-DETR model.

RT-DETR stands as the pioneering Transformer-driven architecture capable of real-time end-to-end inference; its architecture is illustrated in Figure 2. Centered on an anchor-free paradigm, the model avoids the positive-negative sample imbalance and computational redundancy associated with traditional anchor mechanisms through a decoupled detection head for classification and regression, along with an end-to-end inference pipeline. Furthermore, it employs a hybrid encoder that merges CNN-driven local feature acquisition with Transformer-powered global contextual modeling. Combined with a dynamic query selection mechanism, it achieves effective amalgamation of multi-scale representations and adaptive object matching, thereby obtaining an improved trade-off between detection speed and accuracy.

### 3.3. FusionLite-DETR Overview

**Figure 3:**
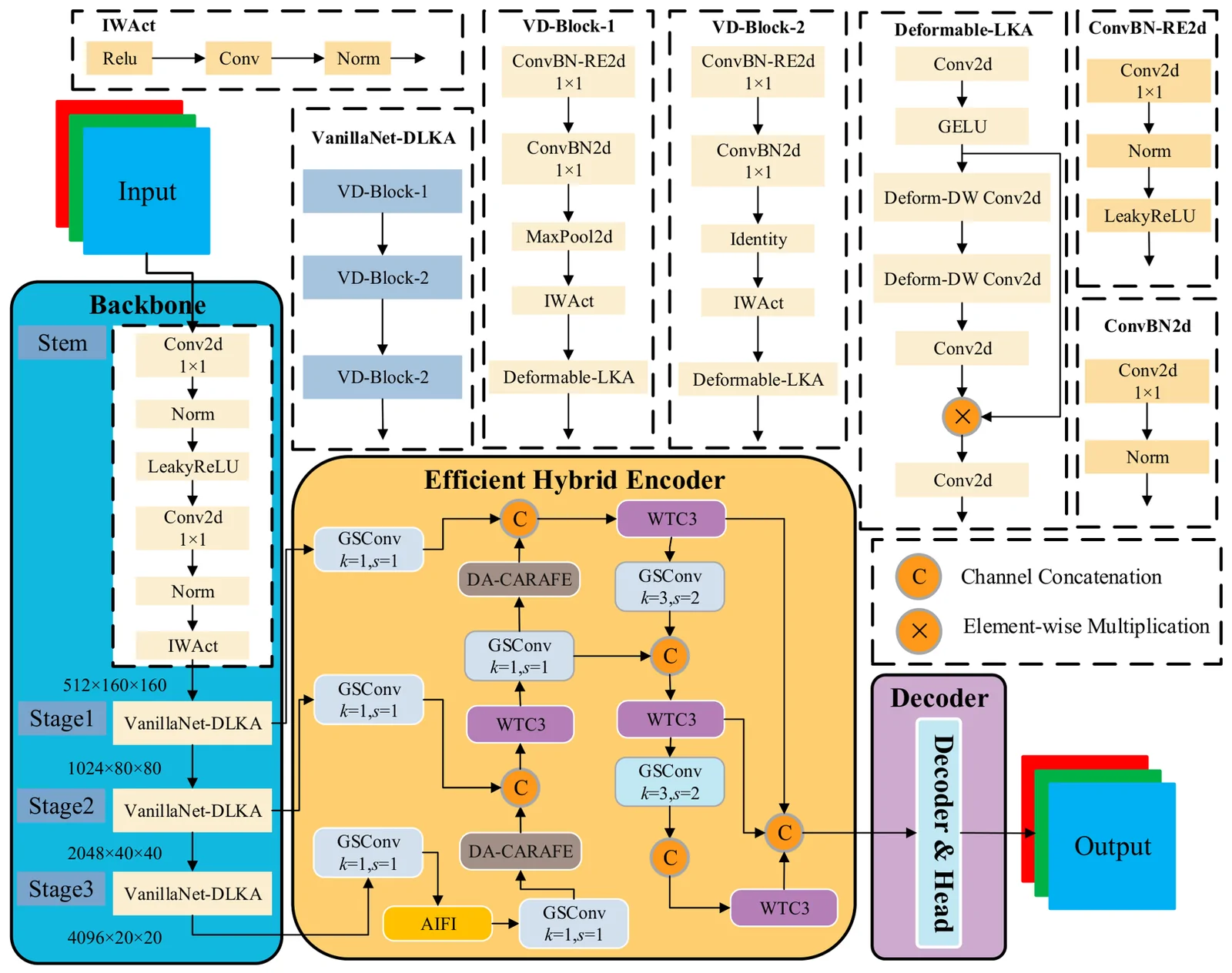
FusionLite-DETR model.

To address the issue of insufficient detection accuracy for transmission line insulator defects in UAV inspections, this study introduces a lightweight, improved RT-DETR model named FusionLite-DETR, the architecture of which is shown in Figure 3. First, VanillaNet-DLKA facilitates lightweight deployment while enhancing the model’s capability to detect small and irregular defects through deformable large-kernel attention. Second, DA-CARAFE reconstructs insulator surface details more accurately than traditional upsampling methods, improving detection sensitivity for subtle defects such as micro-cracks. Meanwhile, the improved feature fusion module, WTC3, enables the simultaneous capture of high-frequency and low-frequency features on insulator surfaces. To further strengthen generalization, GSConv is introduced to reduce redundant computation through channel-shuffling operations, enhancing adaptability to rotation and scale variations. The proposed FusionLite-DETR effectively improves detection accuracy compared to other established models.

### 3.4. VanillaNet-DLKA Backbone Network

**Figure 4:**
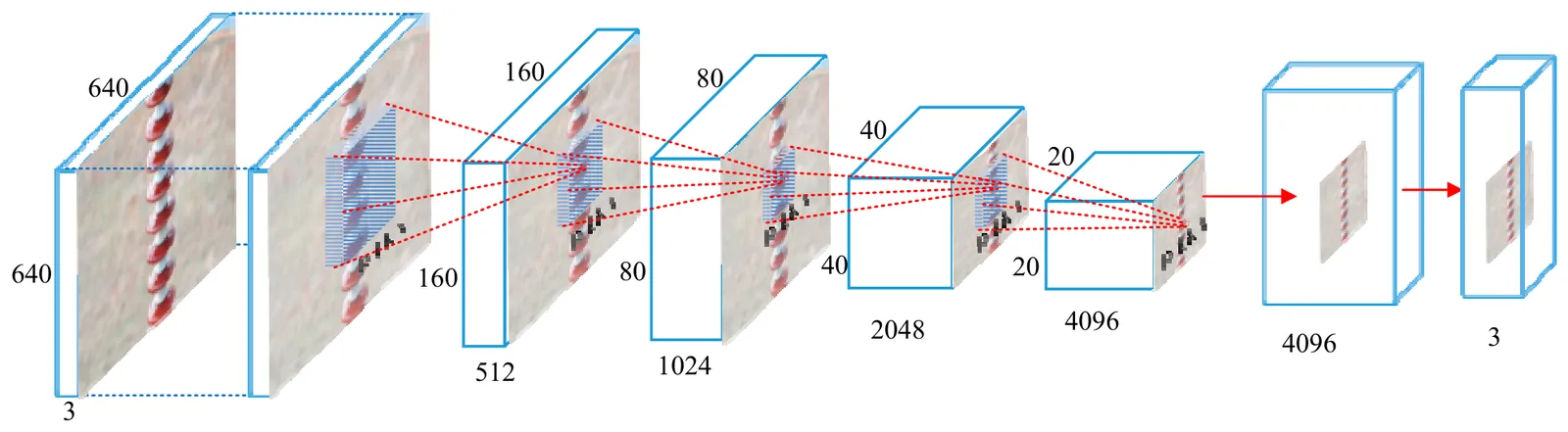
Structure of the VanillaNet-DLKA module.

Transmission line insulator defects typically manifest as elongated objects characterized by large-scale variations, irregular shapes, distribution along the string, and severe background interference. Consequently, the model requires not only a large receptive field and global context modeling capabilities but also high sensitivity to local geometric deformations and crack details. Based on this, we propose a lightweight multi-scale feature extraction backbone network, VanillaNet-DLKA, as illustrated in Figure 4. The VanillaNet-DLKA backbone couples the lightweight, high-throughput convolutional skeleton of VanillaNet with Deformable-LKA modules. The former provides a shallow but high-bandwidth feature transmission path, while the latter implements global modeling approximating self-attention alongside deformable sampling. This design provides a computationally efficient backbone that balances feature-representation capability and inference efficiency, thereby supporting subsequent multiscale feature fusion and the accurate localization of small defects.

VanillaNet abandons residual connections and complex operator designs, resulting in an extremely simplified convolutional neural network architecture. Its forward propagation can be strictly expressed as

(1) $$X_{\mathrm{out}}=\mathrm{Concat}[{\mathrm{Conv}}_{\mathrm{depth}}(X),{\mathrm{Conv}}_{\mathrm{point}}(X)]$$

where ${\mathrm{Conv}}_{\mathrm{depth}}$ represents deep convolution, ${\mathrm{Conv}}_{\mathrm{point}}$ denotes pointwise convolution, and Concat[⋅,⋅] denotes the channel-wise concatenation operation.

Deformable-LKA integrates deformable convolution and large-kernel attention to achieve adaptive modeling of defect geometries. The formula is given by

(2) $$Y(i,j)=\sum_{p=0}^{K-1}\sum_{q=0}^{K-1}w(p,q)X(i+\delta_{x}(i,j,p,q),j+\delta_{y}(i,j,p,q))$$

(3) $$w(p,q)=\frac{\exp(\mathrm{MLP}(X_{pos}(p,q)))}{\sum_{p^{\prime },q^{\prime }}\exp(\mathrm{MLP}(X_{pos}(p^{\prime },q^{\prime })))}$$

where w(p,q) denotes the normalized attention weight for the relative sampling location (p,q); $\delta_{x}$ and $\delta_{y}$ represent learnable offsets for adaptive deformable sampling in defect regions; and $X_{pos}(p,q)$ denotes the corresponding position-aware feature.

### 3.5. DA-CARAFE Upsampling

In the original RT-DETR architecture, nearest-neighbor interpolation is employed at two stages of the top-down feature-fusion pathway to increase the spatial resolution of low-resolution semantic features. This operation follows a fixed sampling rule and does not adapt the reconstruction process to local image content. To enable content-adaptive feature reconstruction and enhance the response to defect-related regions, the two native nearest-neighbor upsampling operations are replaced with the proposed DA-CARAFE.

**Figure 5:**
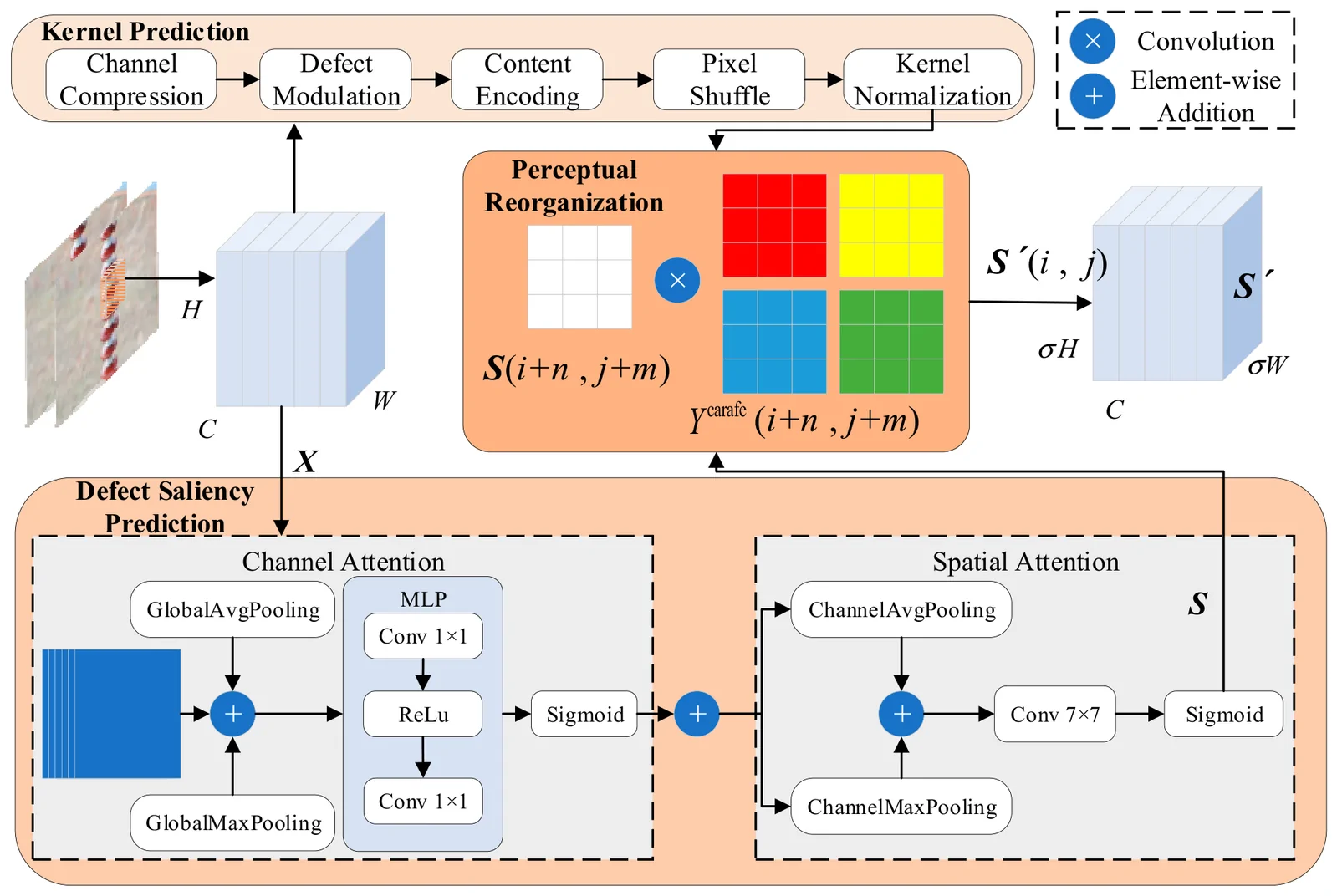
Structure of the DA-CARAFE module.

DA-CARAFE is developed based on the content-aware feature reassembly principle of CARAFE [43]. Unlike the native fixed interpolation operation, it predicts spatially adaptive reassembly kernels from the input features. To further emphasize defect-related information during feature reconstruction, a dual-attention mechanism adapted from the channel and spatial attention modules of convolutional block attention module (CBAM) [44] is introduced to construct a defect saliency map. The resulting saliency map guides both reassembly-kernel prediction and output feature fusion. Consequently, DA-CARAFE reconstructs low-resolution semantic features according to local image content and defect-related responses before their fusion with higher-resolution backbone features. The detailed architecture of DA-CARAFE is illustrated in Figure 5.

Specifically, given an input feature map $X\in {\mathbb{R}}^{B\times C\times H\times W}$, the channel attention map is first generated using global average pooling and global max pooling followed by a shared multilayer perceptron, as defined below:

(4) $$\begin{array}{l} M_{c}=\sigma (\mathrm{MLP}(\mathrm{GAP}(X))+\mathrm{MLP}(\mathrm{GMP}(X)))\\ X_{c}=M_{c}⊙X \end{array}$$

where $M_{c}$ indicates the resulting channel attention map, while $X_{c}$ represents the channel-recalibrated feature map. GAP(·) and GMP(·) denote the Global Average Pooling and Global Max Pooling operations, respectively. MLP(·) refers to a multi-layer perceptron, σ represents the sigmoid activation function, and ⊙ denotes element-wise multiplication.

Following this, a spatial attention mechanism is executed on the channel-recalibrated feature $X_{c}$ to generate the final defect saliency map, as expressed by the following equation:

(5) $$S=\sigma ({\mathrm{Conv}}_{k_{s}\times k_{s}}([{\mathrm{Avg}}_{c}(X_{c}),{\mathrm{Max}}_{c}(X_{c})]))$$

where S denotes the final defect saliency map, and ${\mathrm{Avg}}_{c}(\cdot )$ and ${\mathrm{Max}}_{c}(\cdot )$ refer to the average pooling and max pooling operations along the channel dimension, respectively. ${\mathrm{Conv}}_{k_{s}\times k_{s}}$ represents the convolution operator with a kernel size of $k_{s}\times k_{s}$.

The resulting defect saliency map $S\in {\mathbb{R}}^{B\times 1\times H\times W}$ is subsequently used at two stages of DA-CARAFE. First, S modulates the channel-compressed feature used for reassembly-kernel prediction. Second, its bilinearly resized form $S^{\prime }$ controls the output fusion between the interpolated feature and the CARAFE-reassembled feature. Thus, the same defect saliency information guides both kernel generation and output feature fusion.

In the reassembly-kernel prediction branch, the input feature X is first compressed using a 1×1 convolution to obtain $F_{comp}$. The defect saliency map S is then used to modulate the compressed feature before the reassembly kernel is generated:

(6) $$F_{comp}=C{\mathrm{onv}}_{1\times 1}(X),{\tilde{F}}_{comp}=(1+S)⊙F_{comp}$$

where $F_{comp}\in {\mathbb{R}}^{B\times 1\times H\times W}$ denotes the channel-compressed feature, and ${\tilde{F}}_{comp}$ denotes its saliency-modulated form. The saliency map S is broadcast along the channel dimension during element-wise multiplication.

The saliency-modulated compressed feature is subsequently processed by the kernel encoder and the PixelShuffle [45] operation, after which the predicted weights are normalized using Softmax along the reassembly-kernel dimension to generate spatially adaptive, defect-aware reassembly kernels. These kernels are applied to the local feature patches to obtain the content-aware reassembled feature. In parallel, the input feature is upsampled using nearest-neighbor interpolation to obtain the initial interpolated feature. The defect saliency map is then resized by bilinear interpolation to match the spatial resolution of the interpolated and reassembled features. Finally, the resized saliency map controls the fusion of these two features to produce the final DA-CARAFE output, as expressed below:

(7) $$Y=(1-S^{\prime })⊙U+(1+\lambda_{out}S^{\prime })⊙Y^{\mathrm{carafe}}$$

where Y represents the final fused output of DA-CARAFE, $Y^{\mathrm{carafe}}$ denotes the features obtained from the CARAFE operation, $S^{\prime }$ represents the up-sampled defect saliency map, U is the interpolation result, and $\lambda_{out}$ is the coefficient that controls the enhancement strength of the defect regions. In the main experiments, $\lambda_{out}$ was set to 0.5, and its influence is evaluated through the sensitivity analysis presented in Section 4.5.3.

### 3.6. Feature Fusion Module

#### 3.6.1. GSConv

**Figure 6:**
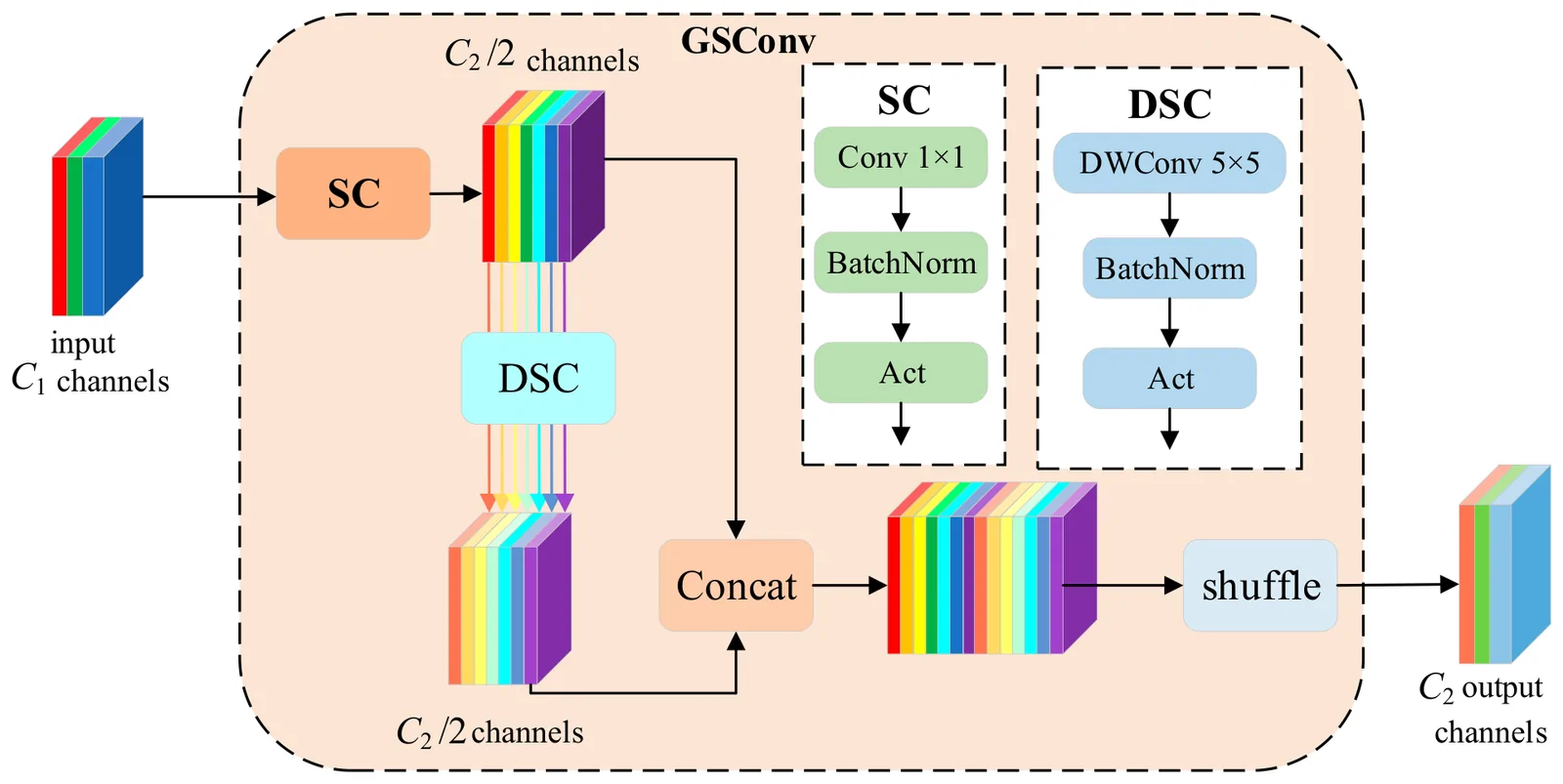
Structure of the GSConv module.

To improve the computational efficiency of the detection network during inference, this paper introduces GSConv as a lightweight convolutional unit in the feature fusion stage to replace the original standard convolution (Conv-BN-Act), as illustrated in Figure 6. In common detection backbone networks, features undergo layer-wise transformations from spatial resolution compression to channel dimension expansion, inevitably leading to the loss of spatial details and semantic information during compression. Standard convolution fully models the implicit correlations between channels but incurs high computational costs, whereas depth-wise convolution reduces computational complexity but weakens channel information interactions. Through a dense convolution branch, a depth-wise convolution branch, and channel shuffle, GSConv preserves inter-channel connections at a lower time complexity, thereby balancing representation capability and efficiency.

Specifically, GSConv first uses standard convolution to generate a portion of the channel features, preserving sufficient semantic representation ability. Then, depthwise convolution is applied to this portion of the features to enhance local spatial modeling. Finally, channel concatenation and channel reordering operations are employed to achieve uniform mixing across branches, as expressed by the following equation:

(8) $$\begin{array}{l} X_{1}=\phi (\mathrm{BN}({\mathrm{Conv}}_{k,s}(X)))\\ X_{2}=\phi (\mathrm{BN}({\mathrm{DWConv}}_{5\times 5}(X_{1})))\\ Y=\mathrm{Shuffle}(\mathrm{Concat}(X_{1},X_{2})) \end{array}$$

where ϕ(·) represents the nonlinear activation function, ${\mathrm{DWConv}}_{5\times 5}$ denotes the depth-wise convolution with a kernel size of 5 × 5, and Shuffle indicates channel reordering.

#### 3.6.2. WTC3

**Figure 7:**
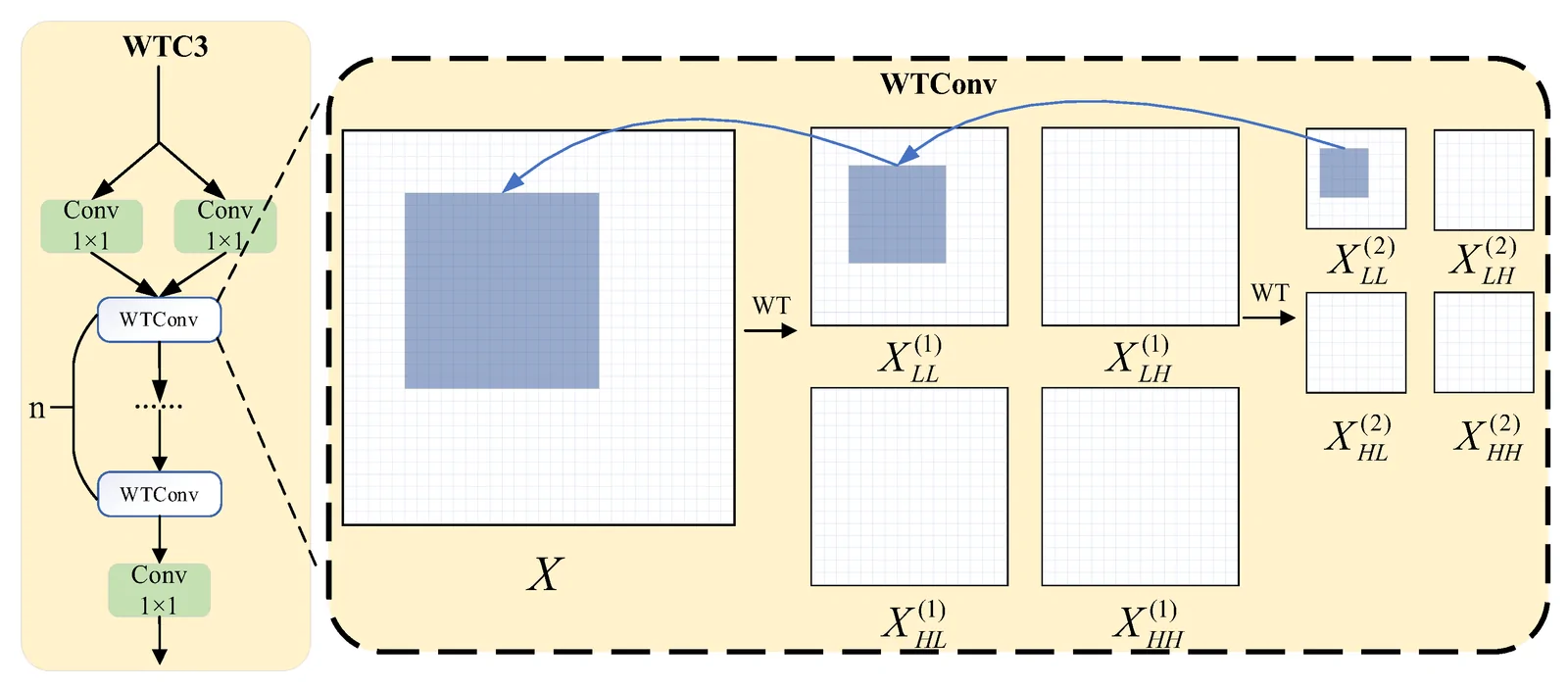
Structure of the WTC3 module.

Surface defects on insulators typically manifest as small cracks, dirt, or wear, which often blend with background noise interference. To overcome this challenge, we introduce a feature fusion module based on WT, named WTC3 (illustrated in Figure 7). This WTC3 unit preserves the structural layout of cross stage partial (CSP) and dual-branch residual fusion but replaces the convolution stack in the main branch with a Discrete Wavelet Transform (DWT)-based Wavelet Transform Convolution (WTConv) stack. First, the input features undergo multi-level wavelet decomposition, yielding a low-frequency component (LL) and three high-frequency sub-bands (LH/HL/HH). Subsequently, separable convolutions are employed to enhance the features within each sub-band, and feature reconstruction is completed through the Inverse Wavelet Transform (IWT). Finally, the reconstructed features are element-wise added to the spatial base branch, explicitly incorporating multi-band information during the feature fusion stage, thereby improving the model’s responsiveness to small targets and weak-texture defects.

The WTC3 module extends the dual-branch residual aggregation form of RepC3 and reconstructs the feature transformation operator in the main branch. A sequence of n WTConv layers replaces the traditional convolution stack. In the feature transformation process, explicit decomposition and multi-band information utilization are applied, transforming the feature fusion from spatial domain local convolution to a joint fusion of both spatial and frequency bands. The process is expressed by the following equation:

(9) $$\begin{array}{l} h_{0}=x\\ h_{i}=W_{i}(h_{i-1}),i=1,2,\cdot \cdot \cdot ,n\\ y=\phi (h_{n}⊕\rho (x)) \end{array}$$

where $W_{i}$ represents the WTConv operator, ϕ(·) denotes the nonlinear activation function, and ρ represents the residual projection.

Specifically, WTConv employs the Haar WT, using a one-dimensional Haar wavelet basis function (with weights $\left[1,1\right]/\sqrt{2}$ and $\left[1,-1\right]/\sqrt{2}$), which is expanded into a two-dimensional space through the outer product of vectors. This process generates four 2 × 2 convolutional kernels, corresponding to the approximation and high-frequency details. The process is expressed by the following equation:

(10) $$f_{LL}=\frac{1}{2}\left[\begin{array}{cc} 1 & 1\\ 1 & 1 \end{array}\right],f_{LH}=\frac{1}{2}\left[\begin{array}{cc} 1 & -1\\ 1 & -1 \end{array}\right],f_{HL}=\frac{1}{2}\left[\begin{array}{cc} 1 & 1\\ -1 & -1 \end{array}\right],f_{HH}=\frac{1}{2}\left[\begin{array}{cc} 1 & -1\\ -1 & 1 \end{array}\right]$$

where $f_{LL}$ represents the low-pass filter, and $f_{LH}$, $f_{HL}$ and $f_{HH}$ represent the high-pass filters.

Due to the quadratic growth of parameter size with the expansion of kernel dimensions, which introduces redundant computations and increases the risk of overfitting, we adopt a frequency band reorganization strategy. First, the input features are decomposed into multiple frequency bands using the WT, and the magnitudes of the low-frequency and high-frequency sub-bands are recalibrated. Then, small kernel depth-wise separable convolutions are applied to the features of each frequency band to perform local modeling. Finally, IWT is applied to reconstruct the features of each sub-band, yielding the final output features Y. This operation is formulated as

(11) Y=IWT(Conv(W,WT(X)))

where X denotes the input tensor, and W indicates the weight tensor of the k × k depth-wise kernel, featuring an input channel count four times that of X.

### 3.7. Inner-MPDIoU Loss Function

Insulator defects are generally small and often exhibit irregular shapes and weak boundary features in UAV images. In such cases, conventional intersection over union (IoU)-based losses, including IoU, Complete IoU (CIoU) [46], and Generalized IoU (GIoU) [47], may remain insufficiently sensitive to fine boundary displacement. Although CIoU incorporates center-distance and aspect-ratio constraints and GIoU extends the overlap measurement to non-overlapping boxes, neither directly constrains the deviations between corresponding box vertices. minimum point distance IoU (MPDIoU) [48] addresses this limitation by introducing the distances between two pairs of corresponding vertices, thereby explicitly measuring the positional discrepancy between the predicted and ground-truth boxes. Therefore, this study replaces the baseline GIoU loss with an Inner-MPDIoU loss that combines the vertex-distance constraint of MPDIoU with the auxiliary-box overlap mechanism of Inner-IoU.

Specifically, given a predicted box $B^{prd}=(x_{1}^{prd},y_{1}^{prd},x_{2}^{prd},y_{2}^{prd})$ and a ground truth (GT) box $B^{gt}=(x_{1}^{gt},y_{1}^{gt},x_{2}^{gt},y_{2}^{gt})$, MPDIoU measures the distances between their corresponding upper-left and lower-right vertices as follows:

(12) $$\begin{array}{l} d_{1}^{2}={\left(x_{1}^{prd}-x_{1}^{gt}\right)}^{2}+{\left(y_{1}^{prd}-y_{1}^{gt}\right)}^{2}\\ d_{2}^{2}={\left(x_{2}^{prd}-x_{2}^{gt}\right)}^{2}+{\left(y_{2}^{prd}-y_{2}^{gt}\right)}^{2} \end{array}$$

The MPDIoU loss is then defined as

(13) $$\begin{array}{l} D_{\mathrm{MPD}}=\frac{d_{1}^{2}}{w^{2}+h^{2}}+\frac{d_{2}^{2}}{w^{2}+h^{2}}\\ L_{\mathrm{MPDIoU}}=1-(IoU-D_{\mathrm{MPD}}) \end{array}$$

where w and h stand for the width and height of the input image, respectively, whereas $d_{1}$ and $d_{2}$ indicate the spatial variations at the upper-left and lower-right vertices.

Inner-IoU [49] constructs auxiliary boxes by concentrically scaling the predicted and ground-truth boxes while keeping their center coordinates unchanged. In the implementation used in this study, the scaling factor is empirically fixed at r = 0.75 throughout training. The dimensions of the auxiliary boxes are as follows:

(14) $$\begin{array}{l} w_{gt}^{in}=w_{gt}\cdot r,h_{gt}^{in}=h_{gt}\cdot r\\ w_{prd}^{in}=w_{prd}\cdot r,h_{prd}^{in}=h_{prd}\cdot r \end{array}$$

Let $\left(x_{c,k},y_{c,k}\right)$, $w_{k}$, and $h_{k}$ denote the center coordinates, width, and height of box k, respectively, where k∈{prd,gt}, with prd and gt representing the predicted box and the ground-truth box, respectively. Since concentric scaling preserves the box center, the boundary coordinates of the corresponding auxiliary box are given by

(15) $$\begin{array}{l} x_{1,k}^{in}=x_{c,k}-\frac{rw_{k}}{2},x_{2,k}^{in}=x_{c,k}+\frac{rw_{k}}{2}\\ y_{1,k}^{in}=y_{c,k}-\frac{rh_{k}}{2},y_{2,k}^{in}=y_{c,k}+\frac{rh_{k}}{2} \end{array}$$

The intersection width and height of the two auxiliary boxes are calculated as

(16) $$\begin{array}{l} w_{I}^{in}=\max(0,\min(x_{2,prd}^{in},x_{2,gt}^{in})-\max(x_{1,prd}^{in},x_{1,gt}^{in}))\\ h_{I}^{in}=\max(0,\min(y_{2,prd}^{in},y_{2,gt}^{in})-\max(y_{1,prd}^{in},y_{1,gt}^{in})) \end{array}$$

Therefore, the intersection area and auxiliary IoU are

(17) $$\begin{array}{c} A_{I}^{in}=w_{I}^{in}h_{I}^{in}\\ IoU_{inner}=\frac{A_{I}^{in}}{r^{2}(w_{gt}h_{gt}+w_{prd}h_{prd})-A_{I}^{in}+\varepsilon } \end{array}$$

where ε is a small positive constant introduced to prevent division by zero. Unlike the original expression based only on the widths and heights of the two boxes, Equations (15)–(17) explicitly account for their center positions and boundary coordinates. It therefore correctly represents the overlap between the two auxiliary boxes.

Finally, the auxiliary-box IoU is incorporated into MPDIoU by replacing the original IoU term while retaining the vertex-distance constraints:

(18) $$L_{\mathrm{Inner}\text{-}\mathrm{MPDIoU}}=1-(IoU_{inner}-D_{\mathrm{MPD}})=L_{\mathrm{MPDIoU}}+IoU-IoU_{inner}$$

The point distance constraint in MPDIoU, combined with the gradient adjustment mechanism of Inner-IoU, allows Inner-MPDIoU to improve boundary box regression while further enhancing the representation of defect region boundaries through the point distance constraint. By replacing the original IoU term with the auxiliary-box IoU while retaining the paired-corner distance constraints, Inner-MPDIoU provides joint supervision for overlap quality and coordinate alignment. The empirical effect of this formulation is evaluated through the scaling-ratio sensitivity study and the loss-function comparison in Section 4.6.

## 4. Results

### 4.1. Dataset

To evaluate model performance on both a public benchmark and real-world industrial inspection data, this study uses two datasets, namely the publicly available Insulator Fault Detection Dataset (IFDD) and the Private Multi-Scenario Insulator Defect Dataset (PMID), the latter of which was supplied by a cooperating power utility company. The category definitions and fixed training, validation, and test partitions described below were used consistently throughout all experiments.

(1) IFDD Dataset

**Figure 8:**
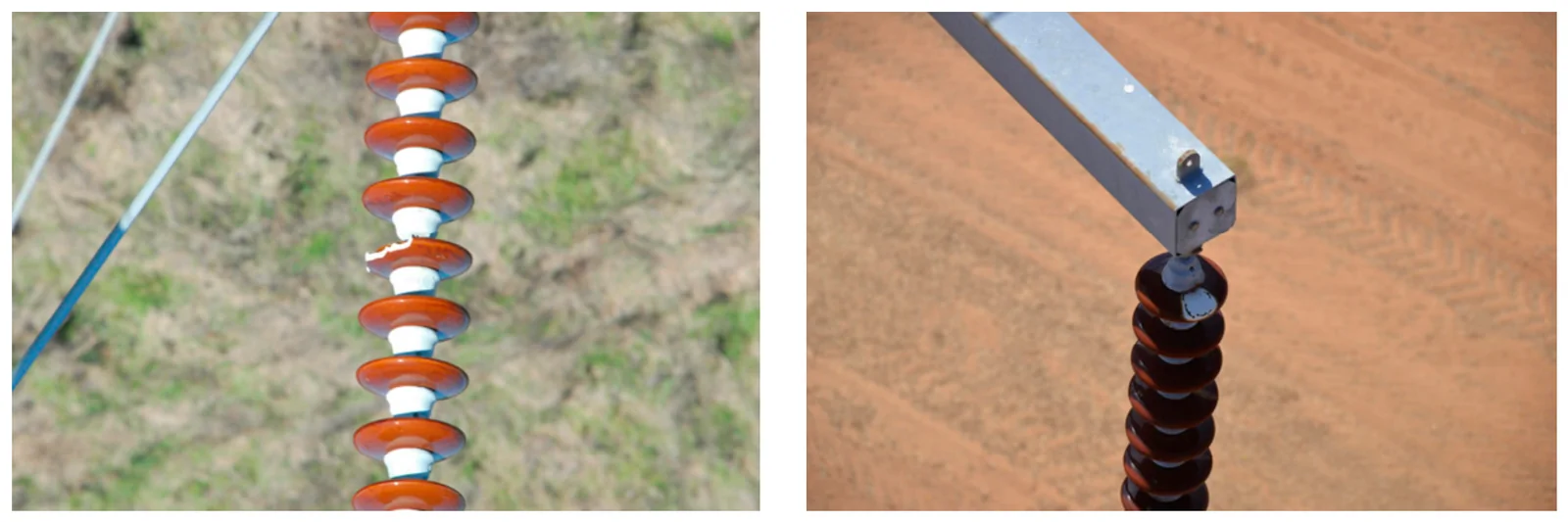
IFDD dataset samples module.

The IFDD dataset contains 1600 inspection images, including 1296 training images, 144 validation images, and 160 test images. It includes three annotated categories: pollution flashover, broken insulator, and intact insulator. The dataset contains 5373 annotated instances, consisting of 2448 pollution-flashover instances, 1093 broken-insulator instances, and 1832 intact-insulator instances. Representative samples are shown in Figure 8.

(2) PMID Dataset

**Figure 9:**
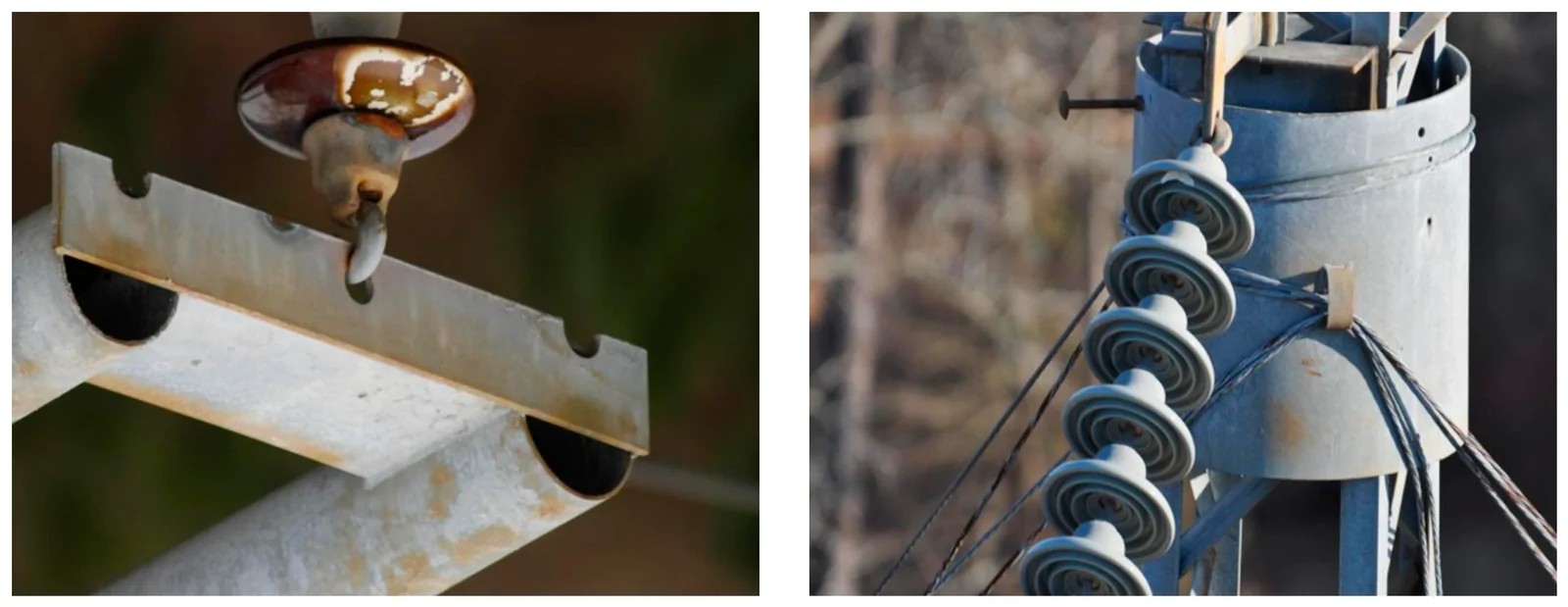
PMID dataset samples module.

The PMID images and corresponding annotations were provided by a cooperating power utility company and originated from routine transmission-line inspection operations. The dataset contains 3110 images, including 2721 training images, 259 validation images, and 130 test images. It comprises two annotated categories: broken insulator and intact insulator. Among its 5591 annotated instances, 2035 are broken insulators, and 3556 are intact insulators. The available data package does not contain metadata linking individual images to specific UAV flights, acquisition sessions, transmission towers, or physical insulators. To assess possible image-level information leakage, all 3110 images were screened for exact duplicates and visually near-duplicate images across the training, validation, and test partitions using exact file hashing and perceptual hashing based on pHash and dHash, with Hamming-distance thresholds of 10 and 6, respectively. No cross-partition duplicate or near-duplicate image was identified under the adopted criteria. Representative samples are shown in Figure 9.

### 4.2. Experimental Environment

To ensure the stability and convergence of the model training process, as well as the reproducibility of experimental results, this study adopts a standardized parameter configuration for the training phase of the insulator defect detection model.

In the input data preprocessing step, all inspection images fed into the model are normalized in resolution, uniformly resized to a fixed dimension of 640 × 640 pixels. For training hyperparameters, the core parameters are determined through preliminary experiments. Specifically, the number of training epochs is set to 200, the batch size is set to 4, the number of data loading worker threads is fixed at 0, the AdamW optimizer is used, and the weight decay coefficient is set to 0.001. All other training parameters are set to the default values of the model’s native framework, ensuring consistency in experimental parameters.

All hyperparameter selections, including $\lambda_{out}$ and r, were based exclusively on validation-set performance. After the final values were fixed, the held-out test sets were used only for final evaluation and were not involved in model selection.

Both the training and evaluation phases are executed on identical hardware and software setups. Table 1 details the exact system specifications, ensuring that our findings remain reliable and fully reproducible.

### 4.3. Evaluation Metrics

To evaluate detection accuracy, model complexity, and inference efficiency, eight metrics were employed: Precision (P), Recall (R), F1-score, mAP50, mAP50:95, Params (M), GFLOPs, and FPS. To ensure a fair comparison, the predictions generated by all models were converted into a unified format and evaluated using identical dataset splits, class mappings, input resolutions, and matching criteria. Precision, Recall, and F1-score were calculated at a fixed confidence threshold of 0.25 and an IoU threshold of 0.50, whereas mAP50 and mAP50:95 were computed from the complete precision–recall curves using the same COCO evaluation API.

Precision (P) represents the proportion of true-positive detections among all predicted positives, whereas Recall (R) represents the proportion of ground-truth instances correctly detected by the model. The F1-score is the harmonic mean of Precision and Recall and provides a balanced measure of detection accuracy and coverage. Average Precision (AP) is calculated from the precision–recall curve for each object category. mAP50 denotes the mean AP across all categories at an evaluation matching *IoU* threshold of 0.50, whereas mAP50:95 averages the AP values over ten IoU thresholds ranging from 0.50 to 0.95 in increments of 0.05. Higher mAP50:95 indicates more accurate object localization under stricter overlap criteria. The mathematical expressions for these metrics are defined as follows:

(19) $$P=\frac{TP}{TP+FP}\times 100\%$$

(20) $$R=\frac{TP}{TP+FN}\times 100\%$$

(21) $${\mathrm{mAP}50}_{IoU=0.5}=\frac{1}{C}\sum_{i=1}^{C}AP_{i}\times 100\%$$

(22) $$F_{1}\text{-}\mathrm{Score}=\frac{2*P*R}{P+R}\times 100\%$$

(23) $$\mathrm{mAP}50:95=\frac{1}{10C}\sum_{t\in \{0.50,0.55,\ldots ,0.95\}}\sum_{i=1}^{C}AP_{c}^{t}\times 100\%$$

where TP denotes the quantity of accurately identified targets, FP is the volume of incorrect positive predictions, and FN indicates the number of missed ground-truth instances. C stands for the total number of target categories, while ${\mathrm{AP}}_{i}$ is the average precision achieved by the i-th class.

Deployment efficiency is evaluated using Params (M), GFLOPs, and FPS. Params (M) denotes the number of learnable parameters in millions; GFLOPs estimates the theoretical computational cost at an input resolution of 640 × 640 pixels; and FPS measures the actual inference throughput under the specified hardware configuration. Lower Params and GFLOPs, together with higher FPS, indicate a more favorable balance between model complexity and inference efficiency.

### 4.4. Comparative Experiments

To assess the detection accuracy and computational efficiency of FusionLite-DETR, comparative experiments were conducted on the proprietary PMID dataset and the public IFDD dataset. The evaluated methods cover representative detection paradigms, including the two-stage Faster R-CNN, one-stage detectors from the YOLO family, and end-to-end Transformer-based detectors from the DETR family. All models were evaluated under the unified experimental and evaluation protocols described in Section 4.2 and Section 4.3, using identical dataset partitions, category mappings, input resolutions, and evaluation settings.

**Figure 10:**
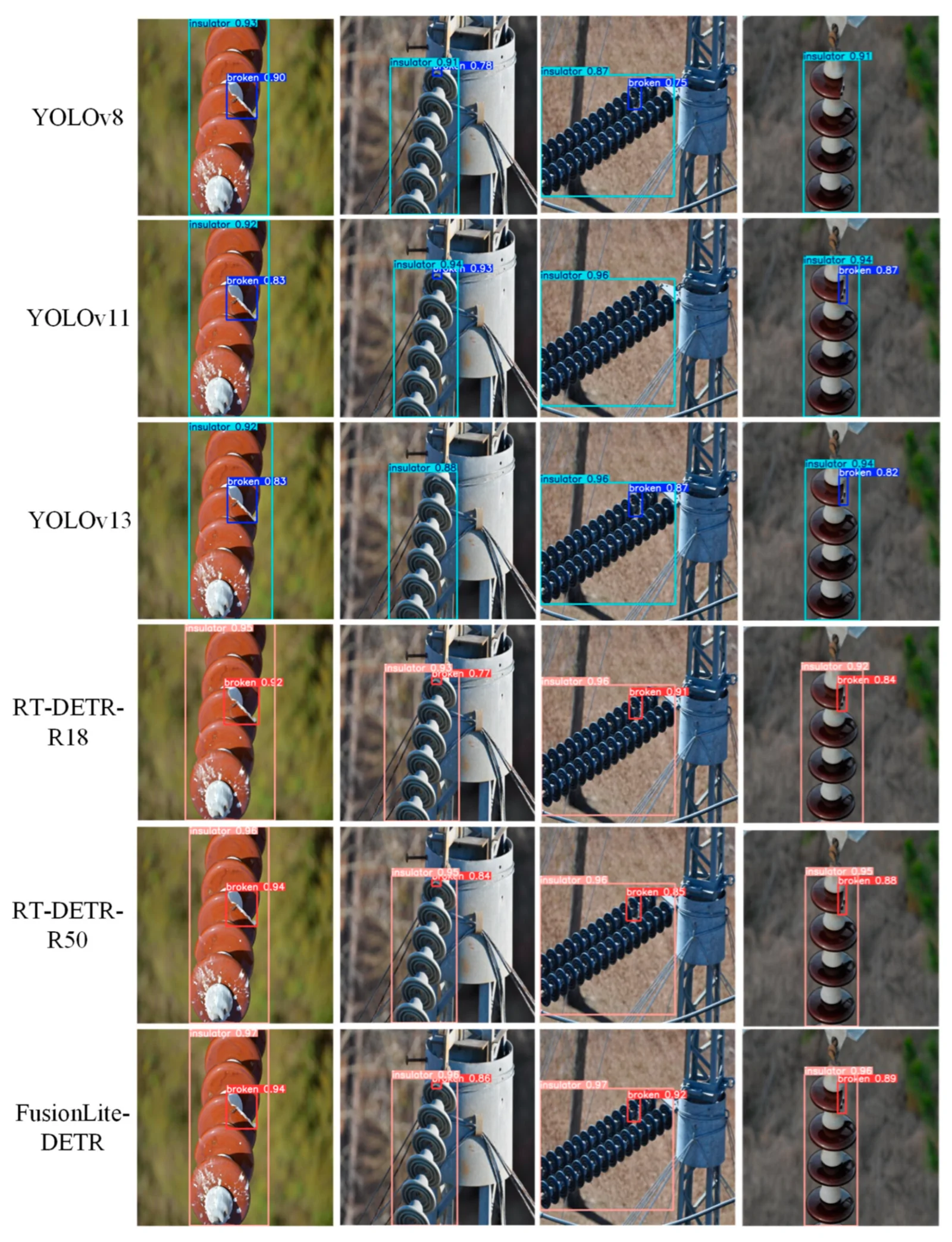
Comparison of different models on the PMID dataset.

Table 2 summarizes the comparative results on the PMID and IFDD datasets. On PMID, FusionLite-DETR achieved a precision of 96.1%, a recall of 93.3%, an F1-score of 94.7%, an mAP50 of 95.5%, and an mAP50:95 of 62.7%. Its mAP50 exceeded those of YOLOv8-M and RT-DETR-R50 by 3.5 and 4.1 percentage points, respectively. Compared with Deformable-DETR and DEIM, FusionLite-DETR achieved improvements of 2.1 and 2.3 percentage points in mAP50, respectively. It also achieved the highest mAP50:95 among the evaluated models on PMID, indicating stronger localization performance across multiple IoU thresholds. Representative detection results are shown in Figure 10.

On the public IFDD dataset, FusionLite-DETR achieved a precision of 92.9%, a recall of 89.8%, an F1-score of 91.3%, an mAP50 of 93.1%, and an mAP50:95 of 61.9%. These results show that FusionLite-DETR maintains competitive detection and localization performance relative to the evaluated one-stage and Transformer-based detectors on the public benchmark. Together with the results obtained on PMID, the IFDD evaluation provides additional evidence of the applicability of the complete architecture across the two investigated datasets.

Regarding model complexity, FusionLite-DETR contains 19.1 M parameters and requires 54.8 GFLOPs at the specified input resolution. Compared with RT-DETR-R50, these values represent reductions of 54.8% in parameter count and 57.4% in computational cost. Under the specified desktop-platform configuration, FusionLite-DETR achieved inference throughputs of 86.3 FPS on PMID and 82.1 FPS on IFDD. Although the proposed model did not achieve the highest inference speed or the lowest computational cost among all evaluated models, it provided a favorable balance among detection accuracy, parameter count, computational cost, and inference throughput under the tested hardware and software configurations.

To provide a category-wise assessment, the AP50 of each annotated category was further examined. On PMID, the AP50 values for broken insulators and intact insulators were 93.20% and 97.77%, respectively. On IFDD, pollution flashover, broken insulators, and intact insulators achieved AP50 values of 87.23%, 92.78%, and 99.34%, respectively. Among the annotated defect categories, pollution flashover exhibited the lowest AP50, indicating that its visual variability and less distinct boundary characteristics present a greater detection challenge. The datasets do not provide finer-grained annotations for specific physical phenomena; therefore, further subtype-level evaluation was not performed.

### 4.5. Ablation Experiments

#### 4.5.1. Component Ablation

Table 3 reports the component-level ablation results on the PMID dataset. The original RT-DETR-R18 serves as the native baseline, achieving a precision of 93.7%, a recall of 88.3%, an mAP50 of 91.6%, and an mAP50:95 of 59.8%. Replacing ResNet-18 with VanillaNet increased precision to 95.0% but reduced recall, mAP50, and mAP50:95 to 83.5%, 88.8%, and 58.6%, respectively. Therefore, the VanillaNet configuration represents the backbone replacement used as the structural basis for the subsequent modifications, rather than an accuracy-improving component by itself.

Integrating DLKAttention into VanillaNet increased precision and recall by 0.8 and 1.9 percentage points, respectively, and increased mAP50:95 from 58.6% to 59.5%, although mAP50 decreased slightly from 88.8% to 88.4%. This result indicates that the contribution of DLKAttention is not uniform across all evaluation metrics. Among the modules evaluated individually with VanillaNet, DA-CARAFE produced only minor metric variations, obtaining an mAP50 of 88.7% and an mAP50:95 of 58.9%. WTC3 reduced mAP50 and mAP50:95 to 88.1% and 57.5%, respectively. In contrast, GSConv provided the greatest standalone improvement, increasing recall to 91.6%, mAP50 to 93.4%, and mAP50:95 to 59.7%.

The combined configurations further demonstrate that the contributions of the proposed modules are configuration-dependent. Adding DA-CARAFE to VanillaNet-DLKA increased recall from 85.4% to 86.2% and mAP50:95 from 59.5% to 60.1%, while mAP50 remained unchanged at 88.4%. Adding DA-CARAFE to the VanillaNet-DLKA-WTC3 configuration increased mAP50 from 87.6% to 90.9% and mAP50:95 from 59.8% to 60.9%. Nevertheless, the performance degradation observed for VanillaNet-DLKA-WTC3 indicates that the results do not support a direct positive pairwise interaction between DLKAttention and WTC3.

The contribution of WTC3 becomes evident in the leave-one-module-out comparison. Adding WTC3 to the otherwise identical VanillaNet-DLKA-DA-CARAFE-GSConv configuration increased precision, recall, mAP50, and mAP50:95 from 95.9%, 92.1%, 93.5%, and 61.4% to 96.1%, 93.3%, 95.5%, and 62.7%, respectively. This comparison suggests that WTC3 provides a conditional and non-redundant contribution within the complete feature-reconstruction and fusion architecture, rather than an independent improvement when directly combined with the backbone.

The complete FusionLite-DETR configuration achieved a precision of 96.1%, a recall of 93.3%, an mAP50 of 95.5%, and an mAP50:95 of 62.7%. Compared with the native RT-DETR-R18 baseline, these values represent increases of 2.4, 5.0, 3.9, and 2.9 percentage points, respectively. The final performance improvement therefore arises from the coordinated operation of the complete architecture rather than from uniformly positive effects of every individual component.

#### 4.5.2. Visualization Analysis of Feature Responses

**Figure 11:**
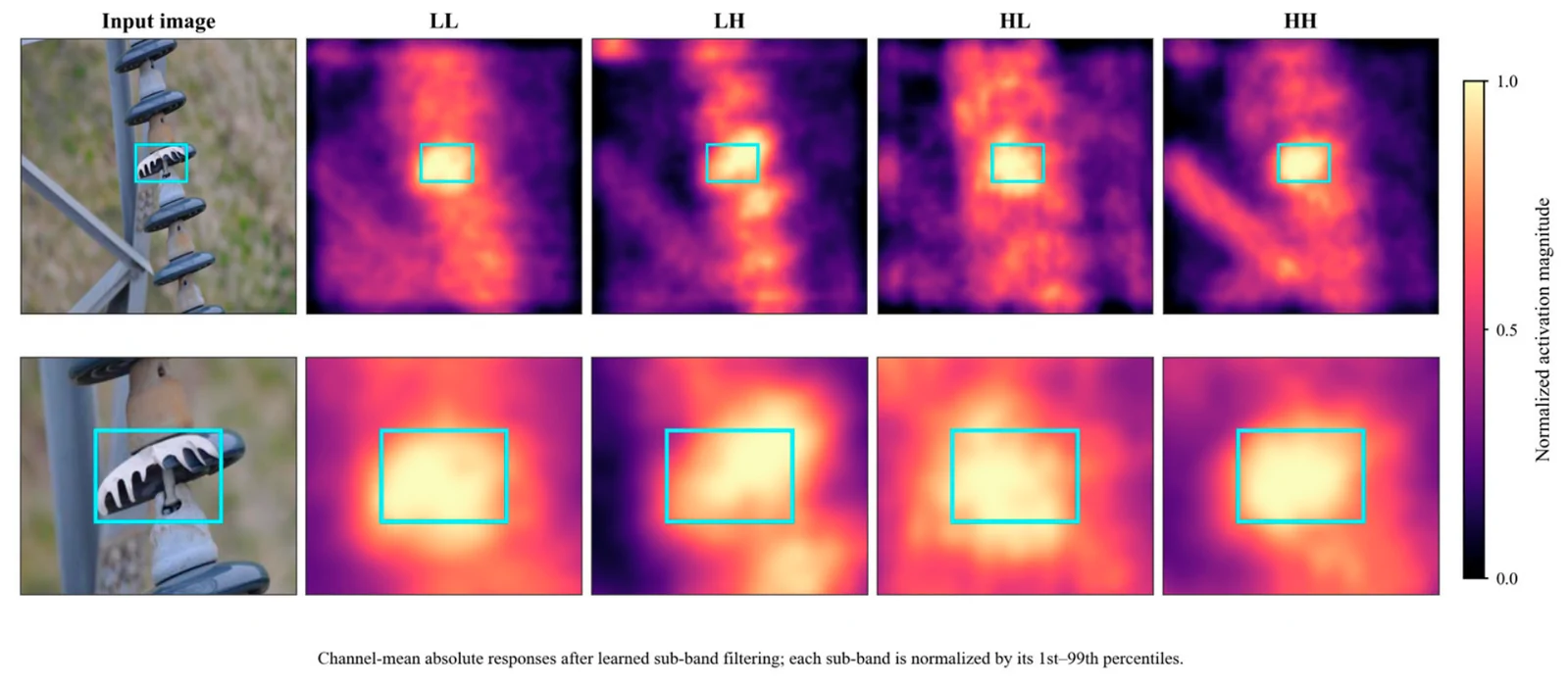
Frequency sub-band activation maps generated by WTC3.

To further examine the feature-processing behavior of the proposed modules, the frequency-sub-band responses of WTC3 and the defect-saliency responses of DA-CARAFE were visualized. Figure 11 presents the LL, LH, HL, and HH sub-band responses generated by WTC3. The blue rectangles indicate the same defect-centered region of interest (ROI) in the input image and the four sub-band response maps, while the second row presents enlarged views of the corresponding boxed regions. Within the blue-boxed region, the LL sub-band retains the overall structure and low-frequency appearance of the insulator, whereas the high-frequency sub-bands emphasize local discontinuities, directional edges, and the irregular boundaries of the broken shed. However, similar responses are also observed along intact insulator contours and other structural edges. This observation indicates that wavelet decomposition preserves local frequency variations but does not independently distinguish defect-related details from semantically irrelevant high-frequency responses, which is consistent with the unfavorable standalone ablation result of WTC3.

**Figure 12:**
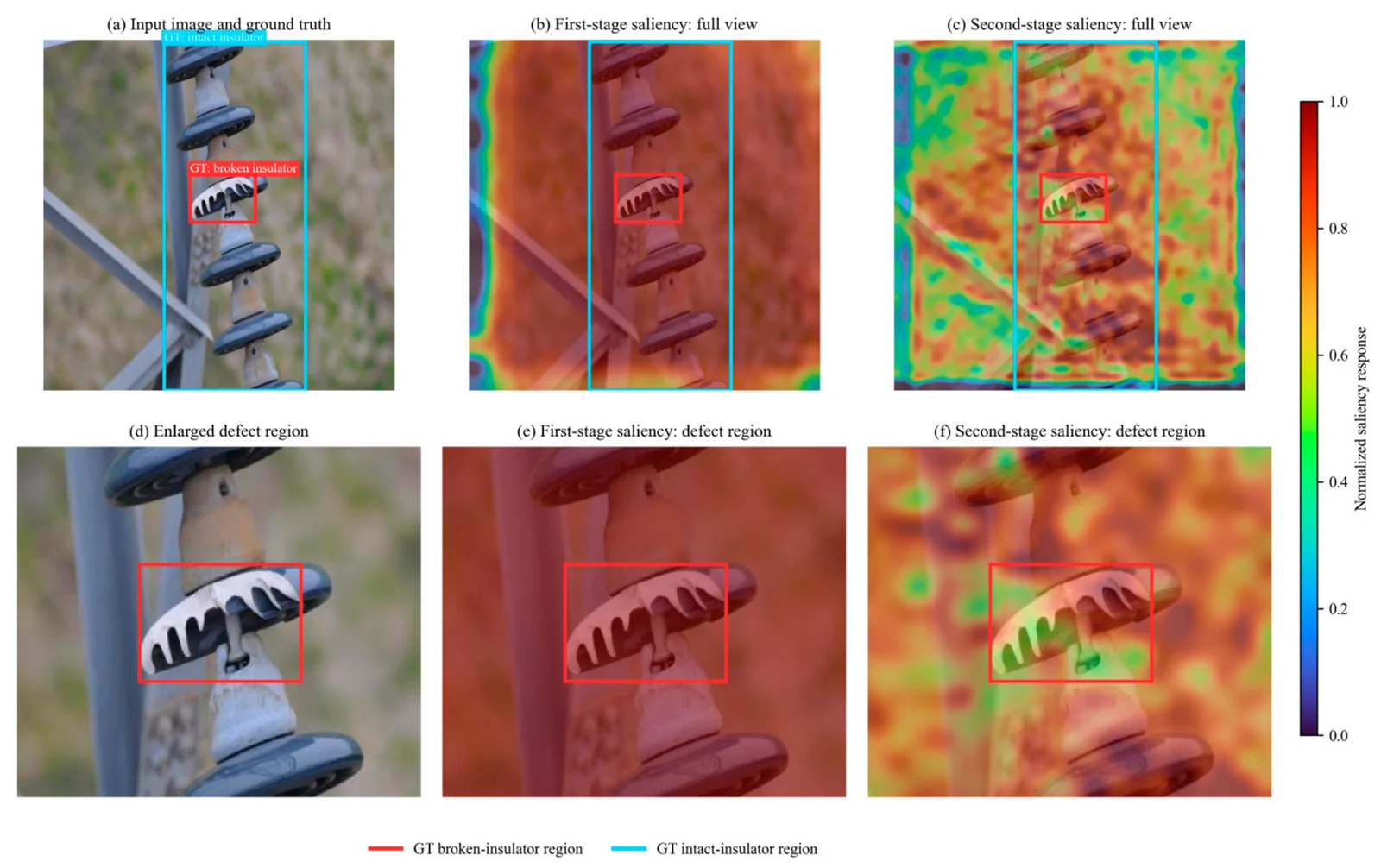
Visualization of the defect-saliency responses generated by DA-CARAFE.

Figure 12 presents the saliency responses generated at the two DA-CARAFE feature-reconstruction stages. The first-stage map produces a relatively broad response over the insulator structure and its surrounding regions, whereas the second-stage map exhibits more spatially differentiated responses around the insulator components and the annotated broken-shed region. The enlarged views further show that the responses overlap the damaged shed and its local structural boundaries, although activations are also retained along intact insulator contours and surrounding structural edges. Therefore, the saliency maps are interpreted as soft spatial modulation signals that guide reassembly-kernel prediction and output feature fusion, rather than as pixel-level defect segmentation results. Each saliency map was independently normalized using its 1st and 99th percentile values for visualization only; this normalization was not involved in model inference.

Taken together, the two visualizations clarify the distinct roles of the modules. WTC3 reorganizes the input features into low- and high-frequency components, while DA-CARAFE performs defect-aware, content-adaptive feature reconstruction and cross-scale alignment. Nevertheless, the visualizations alone do not establish a direct positive interaction between the two modules. Their configuration-dependent contributions are therefore interpreted jointly with the ablation and leave-one-module-out results reported in Table 3.

#### 4.5.3. Sensitivity Analysis of the DA-CARAFE Output Enhancement Coefficient

The coefficient $\lambda_{out}$ controls the strength of the saliency-dependent enhancement applied to the CARAFE-reassembled feature in the output-fusion branch of DA-CARAFE. Because the resized saliency map $S^{\prime }$ is generated by a sigmoid function, its values lie in (0,1), and the corresponding enhancement factor $1+\lambda_{out}S^{\prime }$ is bounded by 1 and $1+\lambda_{out}$. To examine the influence of this coefficient, a one-factor sensitivity analysis was conducted using $\lambda_{out}\in \{0,0.25,0.5,0.75,1.00\}$, representing progressively stronger enhancement of the reassembled feature. The candidate values were compared exclusively on the validation set and were not selected using test-set performance.

For each setting, the complete FusionLite-DETR model was independently trained from scratch using the same dataset partition, initialization seed, input resolution, optimizer, learning-rate schedule, batch size, number of epochs, and data-augmentation strategy. All architectural components and training hyperparameters other than $\lambda_{out}$ were kept unchanged. In the reassembly-kernel prediction branch, the saliency information was directly incorporated according to the formulation described in Section 3.5, without introducing an additional tunable coefficient. It should be noted that setting $\lambda_{out}=0$ does not remove DA-CARAFE or reduce it to conventional CARAFE. It only removes the additional saliency-dependent enhancement applied to the reassembled feature, while the saliency-guided kernel-prediction and interpolation-gating operations remain active.

As shown in Table 4, the evaluated metrics vary within relatively narrow ranges across the tested coefficient values. The mAP50 and mAP50:95 values range from 95.3% to 95.5% and from 62.4% to 62.7%, respectively, indicating limited sensitivity to moderate variations in the output enhancement strength. The setting $\lambda_{out}$ = 0.50 was retained as a representative moderate configuration because it produced the highest observed F1-score, mAP50, and mAP50:95. However, given the small numerical differences among the tested settings, this result is not interpreted as establishing a statistically superior coefficient value.

### 4.6. Loss Function Comparison Experiment

#### 4.6.1. Sensitivity Analysis of the Auxiliary-Box Scaling Ratio

The auxiliary-box scaling ratio r controls the spatial extent of the auxiliary boxes used to calculate Inner-IoU. To examine the sensitivity of Inner-MPDIoU to this hyperparameter, r was varied over {0.50, 0.65, 0.75, 0.85, 1.00, 1.15}. The candidate values of r were compared exclusively using validation-set performance and were fixed before the held-out test sets were evaluated. For each setting, the complete FusionLite-DETR model was independently trained on the PMID and IFDD datasets under the same data split, initialization seed, and training configuration. All architectural components and training hyperparameters other than r were kept unchanged.

Table 5 presents the performance variation under different auxiliary-box scaling ratios. When r varied from 0.50 to 1.15, the PMID dataset showed variations of 0.3 and 0.3 percentage points in mAP50 and mAP50:95, respectively. On the IFDD dataset, the corresponding variations were 0.2 and 0.3 percentage points. The relatively narrow performance ranges indicate that the proposed Inner-MPDIoU loss maintains stable detection performance under moderate changes in the scaling ratio. Considering the comparable results obtained across the tested settings, r = 0.75 was retained as the default value in the subsequent experiments.

#### 4.6.2. Comparison of Bounding-Box Regression Losses

Table 6 presents the comparison of different bounding-box regression losses on the PMID dataset. With the Inner-MPDIoU loss, FusionLite-DETR achieved an F1-score of 94.7%, an mAP50 of 95.5%, and an mAP50:95 of 62.7%. Compared with GIoU, the F1-score and mAP50 increased by 1.3 and 2.8 percentage points, respectively, while the corresponding improvements over SIoU [50] were 0.8 and 1.9 percentage points. These results demonstrate that Inner-MPDIoU provides more effective localization constraints under the present experimental settings. By jointly modeling auxiliary-box overlap and paired-corner distances, the proposed loss imposes complementary constraints on overlap quality and coordinate alignment, thereby providing refined regression supervision for small-scale defects and cases with limited predicted–ground-truth overlap.

## 5. Conclusions

To address the challenges of UAV-based power-line inspection, including small defect regions, complex backgrounds, and weak visual features, we propose FusionLite-DETR, an end-to-end lightweight object detection framework. Through a series of architectural and loss-function improvements, the proposed model achieves a favorable balance between detection accuracy, model complexity, and computational efficiency under the tested desktop-platform configuration.

Experimental results show that FusionLite-DETR achieved mAP50 scores of 95.5% and 93.1% and mAP50:95 scores of 62.7% and 61.9% on the proprietary PMID and public IFDD datasets, respectively. On PMID, its mAP50 was 4.1 percentage points higher than that of RT-DETR-R50, while the results on IFDD remained competitive among the evaluated detectors. FusionLite-DETR contains 19.1 M parameters and requires 54.8 GFLOPs, representing reductions of approximately 54.8% and 57.4%, respectively, relative to RT-DETR-R50. Under the specified desktop-platform configuration, the model achieved inference throughputs of 86.3 FPS on PMID and 82.1 FPS on IFDD. These results indicate a favorable balance among detection accuracy, model complexity, computational cost, and inference throughput under the tested hardware and software configuration.

Future work will focus on the real-device deployment and system-level evaluation of the complete three-terminal collaborative architecture, including UAV-based image acquisition and transmission, service-end model inference, and client-side result visualization. The evaluation will further consider end-to-end latency, communication overhead, memory consumption, and power consumption under practical inspection conditions.

## Figures and Tables

**Table 1:** Experimental environment configuration.

Name	Configuration
Operating System	Windows 11
GPU	NVIDIA GeForce RTX 4060 Laptop GPU
CPU	Intel(R) Core(TM) i7-13650HX
Memory (GPU)	8G
Python Version	3.10
Python Framework	torch-2.6.0+cu126

**Table 2:** Comparative results of different detection models on the PMID and IFDD datasets.

Dataset	Model	P/%	R/%	F1-Score/%	mAP50/%	mAP50:95/%	Params (M)	GFLOPs	FPS
PMID	Faster R-CNN	70.1	65.2	67.5	69.7	41.1	40.5	173.3	22.5
	YOLOv5-M	90.5	83.0	86.6	88.4	56.8	21.2	53.9	31.6
	YOLOv8-M	92.6	90.4	91.5	92.0	60.6	25.9	81.3	82.2
	YOLOv10-M	92.1	83.9	87.8	88.0	58.4	19.8	59.8	54.8
	YOLOv11-L	92.3	85.3	88.7	88.4	59.2	25.3	85.6	51.0
	YOLOv13-L	92.6	87.5	90.0	91.1	59.4	27.6	91.2	56.9
	Deformable-DETR	93.1	89.6	91.3	93.4	61.2	39.8	169.1	19.1
	DINO	93.9	89.2	91.5	92.2	61.0	46.6	62.7	74.5
	DEIM	94.2	91.2	92.7	93.2	61.8	21.4	38.4	70.0
	RT-DETR-R18	93.7	88.3	90.9	91.6	59.8	20.8	56.9	88.6
	RT-DETR-R50	94.0	89.0	91.4	91.4	60.6	42.3	128.6	90.1
	FusionLite-DETR	96.1	93.3	94.7	95.5	62.7	19.1	54.8	86.3
IFDD	YOLOv5-M	85.6	84.6	85.0	87.1	58.4	21.2	53.9	30.8
	YOLOv8-M	90.7	86.9	88.7	89.4	58.6	25.9	81.3	81.3
	YOLOv10-M	83.2	81.7	82.4	83.8	56.9	19.8	59.8	54.1
	YOLOv11-L	90.4	87.2	88.7	91.1	59.7	25.3	85.6	49.6
	YOLOv13-L	91.3	87.9	89.5	92.2	60.8	27.6	91.2	53.8
	Deformable-DETR	90.9	86.8	88.8	92.1	61.1	39.8	169.1	19.0
	DINO	91.2	87.6	89.4	91.5	61.2	46.6	62.7	73.1
	DEIM	91.5	87.9	89.7	92.4	61.4	21.4	38.4	68.9
	RT-DETR-R18	90.8	87.5	89.1	92.0	61.0	20.8	56.9	87.2
	RT-DETR-R50	91.4	88.7	90.0	93.0	61.5	42.3	128.6	88.4
	FusionLite-DETR	92.9	89.8	91.3	93.1	61.9	19.1	54.8	82.1

**Notes:** All models were evaluated with the same protocol (mAP50, mAP50:95, and P/R at confidence 0.25, IoU = 0.5, 640 × 640).

**Table 3:** Ablation study of the backbone and feature-enhancement modules on the PMID dataset.

Backbone	DA-CARAFE	WTC3	GSConv	P/%	R/%	mAP50/%	mAP50:95/%	Params (M)	GFLOPs
RT-DETR-R18 (baseline)				93.7	88.3	91.6	59.8	20.8	56.9
VanillaNet				95.0	83.5	88.8	58.6	16.3	44.6
VanillaNet-DLKA				95.8	85.4	88.4	59.5	17.9	48.5
VanillaNet	√			95.1	84.3	88.7	58.9	16.8	46.3
VanillaNet		√		92.5	81.5	88.1	57.5	17.1	47.2
VanillaNet			√	95.3	91.6	93.4	59.7	16.8	44.9
VanillaNet-DLKA	√			95.9	86.2	88.4	60.1	18.1	51.5
VanillaNet-DLKA		√		93.3	83.4	87.6	59.8	18.3	52.3
VanillaNet-DLKA			√	95.6	91.9	93.4	60.7	17.5	49.2
VanillaNet-DLKA		√	√	95.8	91.7	93.8	61.3	18.8	53.1
VanillaNet-DLKA	√		√	95.9	92.1	93.5	61.4	18.4	51.8
VanillaNet-DLKA	√	√		96.0	85.2	90.9	60.9	18.7	54.5
VanillaNet-DLKA	√	√	√	96.1	93.3	95.5	62.7	19.1	54.8

**Notes:** “√” indicates that the corresponding module is included in the model configuration, whereas a blank cell indicates that the module is not included.

**Table 4:** Sensitivity analysis of the DA-CARAFE output enhancement coefficient on the PMID dataset.

$\lambda_{out}$	P/%	R/%	F1-Score/%	mAP50/%	mAP50:95/%
0.00	95.7	93.0	94.3	95.3	62.4
0.25	96.0	93.1	94.5	95.4	62.5
0.50	96.1	93.3	94.7	95.5	62.7
0.75	95.9	93.3	94.6	95.4	62.6
1.00	95.9	93.2	94.5	95.4	62.5

**Table 5:** Effect of the auxiliary-box scaling ratio on detection performance.

r	PMID mAP50/%	PMID mAP50:95/%	IFDD mAP50/%	IFDD mAP50:95/%
0.50	95.3	62.6	92.9	61.6
0.65	95.3	62.7	93.0	61.7
0.75	95.5	62.7	93.1	61.9
0.85	95.4	62.5	93.0	61.8
1.00	95.3	62.4	93.0	61.8
1.15	95.2	62.4	92.9	61.7

**Table 6:** Loss function comparison experiment on the PMID dataset.

Model	IoU	F1-Score/%	mAP50/%	mAP50:95/%
FusionLite-DETR	GIoU	93.4	92.7	61.0
	CIoU	93.8	93.3	60.3
	EIoU	94.0	93.8	60.5
	SIoU	93.9	93.6	60.4
	InnerIoU	93.1	94.2	61.6
	MPDIoU	94.1	94.6	61.9
	Inner-MPDIoU (r = 0.75)	94.7	95.5	62.7

## Data Availability

The public IFDD dataset is available from its original source at https://doi.org/10.6084/m9.figshare.21200986. The PMID dataset was provided by a cooperating power utility company for use in this study. Owing to data-ownership and confidentiality restrictions, the PMID dataset cannot be publicly released, redistributed, or shared with third parties. Any future access would require authorization from the data owner.

## Abbreviations

The following abbreviations are used in this manuscript:

UAV	Unmanned Aerial Vehicle
CNN	Convolutional Neural Network
DETR	Detection Transformer
IoU	Intersection over Union
FPS	Frames Per Second
PMID	Private Multi-Scenario Insulator Defect Dataset
IFDD	Insulator Fault Detection Dataset
